# Mir-30b-3p affects the migration and invasion function of ovarian cancer cells by targeting the CTHRC1 gene

**DOI:** 10.1186/s40659-020-00277-4

**Published:** 2020-03-10

**Authors:** Yan Li, Jinhua Zhou, Juan Wang, Xiaoping Chen, Yan Zhu, Youguo Chen

**Affiliations:** 1 Department of Obstetrics and Gynecology, The First Affiliated Hospital of Soochow University, 188 Shizi Road, Suzhou, 215006 Jiangsu People’s Republic of China; 2 Department of Obstetrics and Gynecology, The First People’s Hospital of Yancheng, Yancheng, 224001 Jiangsu People’s Republic of China

**Keywords:** miR-30b-3p, Ovarian cancer, OVCAR3, CTHRC1, EMT

## Abstract

**Background:**

The aim of this study was to investigate the effect role and mechanism of miR-30b-3p on ovarian cancer cells biological function.

**Methods:**

The expression of miR-30b-3p was detected in ovarian cancer cell lines and normal ovarian epithelial cell line by qRT-PCR. Mir-30b-3p mimic was transfected into OVCAR3 cells. Cell-counting kit-8 (CCK-8) assay was conducted to explore the effect of mir-30b-3p on the OVCAR3 cells’ proliferation. Cell cycle and apoptosis were detected by Flow cytometry. Cell invasion ability was detected by Transwell test. The regulation of putative target of miR-30b-3p was verified by double luciferase reporter assays and Western blot.

**Result:**

We found that miR-30b-3p was downregulated in OVCAR3 cells. Overexpression of miR-30b-3p suppressed proliferation, promoted apoptosis, slowed cell cycle and inhibited migration and invasion of OVCAR3 cells. Bioinformatics analysis identified 3′-untranslated region (3′UTR) of Collagen triple helix repeat-containing 1 (CTHRC1) as the presumed binding site for miR-30b-3p. Detection of double luciferase reporter and Western-Blot result confirmed that CTHRC1 was the target gene of miR-30b-3p. Furthermore, E-cadherin, β-cadherin and Vimentin protein expression level were changed after transfection of miR-30b-3p.

**Conclusion:**

miR-30b-3p function as an anti-cancer gene. Overexpression of miR-30b-3p can inhibit the biological function of ovarian cancer cells. MiR-30b-3p targets CTHRC1 gene plays an important role in epithelial–mesenchymal transformation (EMT), and supports miR-30b-3p as a potential biological indicator for ovarian cancer in the future.

## Background

Ovarian cancer is one of the three major gynecological malignancies, with about 240,000 new cases and 150,000 deaths worldwide every year [1]. It is the most common cause of gynecological malignancy death. The tragic outcomes of ovarian cancer are mainly late diagnosis as it is generally lack of obvious symptoms [2]. The 5-year survival rate for FIGO stage Ι patients with ovarian cancer is as high as 90%, while the 5-year survival rate for stage III or IV patients remains at less than 30% [3]. Hence, it is urgent to provide more reliable prognosis biomarker to effectively diagnose early and evaluate the prognosis of ovarian cancer. Currently, common clinical markers of ovarian cancer include CA125, CA153 and CEA, but the specificity and sensitivity of these markers are low.

It is the basis of diagnosis and treatment of ovarian cancer to find out the pathogenesis of ovarian cancer from the genetic approach. MicroRNA (miRNA) is endogenous small non-coding RNA with a length of 19 to 25 nucleotides, which can regulate target gene expression by binding to 3′UTR [4]. MiRNAs are involved in many cancer-related biological processes, including tumor genesis, cell proliferation, differentiation and apoptosis, angiogenesis, invasion and metastasis, tumor resistance, and prognosis [5]. Moreover, emerging evidence suggests that miRNAs are present not only in cell but also in circulating blood, reflecting the conditions of tissue or organ [6, 7]. It has become increasingly important to study the mechanism of miRNA’s influence on tumorigenesis.

The miRNA-30 family includes miR-30a, miR-30b, miR-30c-1, miR-30c-2, miR-30d, miR-30e, encoded by six genes located on human chromatids 1, 6, and 8 [8]. It has been reported that miR-30 family express disorders in lung cancer, breast cancer, multiple myeloma, colorectal cancer, liver cancer, bladder cancer, endometrial cancer and other cancers [8, 9]. Furthermore, recent evidence has demonstrated that miR-30 families can act on the cell signaling pathway of corresponding target genes and affect the development, metastasis, apoptosis and drug resistance of ovarian cancer cells, which is expected to be a potential biomarker and therapeutic target of ovarian cancer. However, the regulatory mechanism of miR-30b-3p in response to ovarian cancer remain unclear. The current study was performed with the aim of investigating the effect and mechanism of miR-30b-3p on the biological function of ovarian cancer cells. To evaluate the potential of miR-30b-3p as a biomarker of ovarian cancer, the expression level of miR-30b-3p in ovarian cancer cell were analyzed and compared with those of normal ovarian epithelial cells. We analyzed the effect of mir-30b-3p on the proliferation, cell cycle, migration and invasion of ovarian cancer cells, and investigated whether this effect was related to the CTHRC1.

## Methods

### Materials

Human ovarian cancer epithelial cell line OVCAR3 and human normal ovarian epithelial cell line IOSE80 were purchased from ATCC cell bank in the United States. Cell culture reagents (DEME medium, fetal bovine serum, streptomycin penicillin, trypsin) were purchased from Gibco, USA. Cell proliferation activity assay Kit CCK-8 was purchased from Tongren Institute of Chemistry, Japan. miRNA extraction kit, miRNA reverse transcription and fluorescence quantitative kit, and Lipofectamine TM 2000 transfection kit were all purchased from Invitrogen, USA. Mir-30b-3p, mimics and mimic control miRNAs were synthesized by Shanghai Gemar Pharmaceutical Technology Co., LTD., China. Mir-30b-3p and U6 primers were designed and synthesized by bioengineering (Shanghai) Co., LTD., China. ECL chemiluminescence reagent and BCA protein concentration detection kit were purchased from Shanghai Biyuntian Biotechnology Co., LTD., China. The dual luciferase report detection system is the product of Promega, USA.

### Cell culture

After resuscitation of human ovarian cancer epithelial cell line OVCAR3 and human normal ovarian epithelial cell line IOSE80, DEMN medium containing 10% fetal bovine serum and 1% dual antibody was used for culture, and incubated at 5% CO_2_ and 37 °C. The medium was changed once a day. Digestion, passage and inoculation were carried out after the degree of cell fusion > 80% for follow-up research.

### Quantitative reverse transcription polymerase chain reaction (qRT-PCR) of miR-30b-3p

OVCAR3 cells and IOSE80 cells were collected at logarithmic growth stage. Total RNA was extracted and transcribed back into cDNA. mir-30b-3p expression was detected using U6 as the internal reference The forward and reverse primers for miR-30b-3p were 5′-GCTGCGGTGTAGACATCTAATAC-3′ and 5′-ATCCAGTGCAGGGTCCGACC-3′, and for U6 were 5′-CTCGCTTCGGCAGCACA-3′ and 5′-AACGCTTCACGAATTTGCGT-3′. The relative expression of mir-30b-3p was calculated according to 2^−ΔΔCt^, and expression was shown relative to U6.

### Cell transfection

OVCAR3 cells at logarithmic growth stage were digested and collected. Inoculate in six-well plates at a density of 10^6^/well. After 24 h culture, mir-30b-3p mimic and mimic control were transfected into OVCAR3 cells using Lipofectamine™ 2000 transfection reagent according to the kit operation protocol.

### Cell proliferation was detected using the CCK-8 assay

Human ovarian cancer OVCAR3 cells were further cultured after transfection with mir-30b-3p mimic and no-load plasmid, and the proliferation and activation capacity of the cells were measured after 48 h, respectively. Cell were harvested and seeded into 96-well plates at a density of 10^4^ cells/well. Each group of cells was set up with 3 repeating holes. Following incubation of cell for 0, 24, 48, and 72 h, the CCK-8 reagent of 20 μL was add to each well. The OD490 values was detected by an automatic enzyme marker, which was assessed the number of viable cell.

### Flow cytometry was used to detect cell cycle and apoptosis

Cells were stained with propidium iodide (PI) and the cell cycle distribution and apoptosis rate were detected by flow cytometry. Firstly, the dye PI [containing 50 mg/L, 20 mg/L ribonuclease (RNase) A, 1 g/L sodium citrate, pH 7.4] was prepared, and stored at 4 °C away from light. Two groups of cells were cultured for 48 h after transfection, the cells were washed with phosphate buffer solution (PBS) and fixed with 75% ethanol, discarded supernatant after centrifugal. Precooled PBS was added, and the supernatant was discarded after centrifuged again, and then collected the cells. RNase A reagent was added to digest the cells for 30 min, and then PI solution was added for staining for 30 min under 4 °C. Samples were transferred to the detection tube of flow cytometer for detection. Cell cycle distribution and apoptosis rate were measured according to the changes of DNA content at various stages of cell mitosis.

### Cell migration and invasion was detected by Transwell assay

The upper chamber of the Transwell plate was coated with Matrigel of 1 mg/mL, and DMEM medium was added to the lower chamber. 2 × 10^5^ OVCAR3 cells were seeded in an upper chamber and cultured at indoor temperature. Cells migrated or infiltrated into the lower chamber were fixed and stained with crystal violet. Randomly selected fields were placed under the microscope for counting.

### The target gene of mir-30b-3p was verified by double luciferase activity assay

Wild-type and mutant CTHRC1 double luciferase reporter plasmids were constructed and named CTHRC1-wt3′-UTR and CTHRC1-mut3′-UTR, respectively. 1× Passive Lysis Buffer 300 μL was used to lyse the cells. 40 μL cell lysate was absorbed into the Lockwell maxisorp Assay plate, added 20 μL luciferase Assay Reagent, and the fluorescence value of luciferase was measured by elisa immediately after shaking and mixing. After adding 20 μL Stop & Glo^®^ Reagent for each well, use the enzyme marker to detect the fluorescence value of sea kidney luciferase.

### Detection of CTHRC1 protein expression in OVCAR3 cells

The expression of CTHRC1 was detected by Western blot. The cells were lysed in RIPA buffer, and the concentrations of cell proteins were determined according to the BCA kit. Samples were denatured by boiling for 5 min. 40 μg total protein separated on 10% SDS-PAGE (70 V, 30 min; 100 V, 90 min) and transfer to PVDF membrane (200 mA, 3 h). Membranes were then blocked in 5% non-fat milk and incubated with primary antibody (1:500) overnight at 4 °C. The next day, the membrane was washed with TPBS and PBS and incubated with secondary antibodies. And then performed Western blot.

### Statistical analysis

The potential target genes of miR-30b-3p were identified by an online tool of miRNA target prediction database (Trargetscan.org).

All experimental results were statistically analyzed by SPSS 18.0 or Graph prism software. Measurement data are expressed as mean ± standard. t test was used to compare the two groups and analysis of variance was used to compare the three groups. P < 0.05 was considered statistically significant.

## Result

### The expression level of miR-30b-3p in ovarian cells

**Fig. 1:**
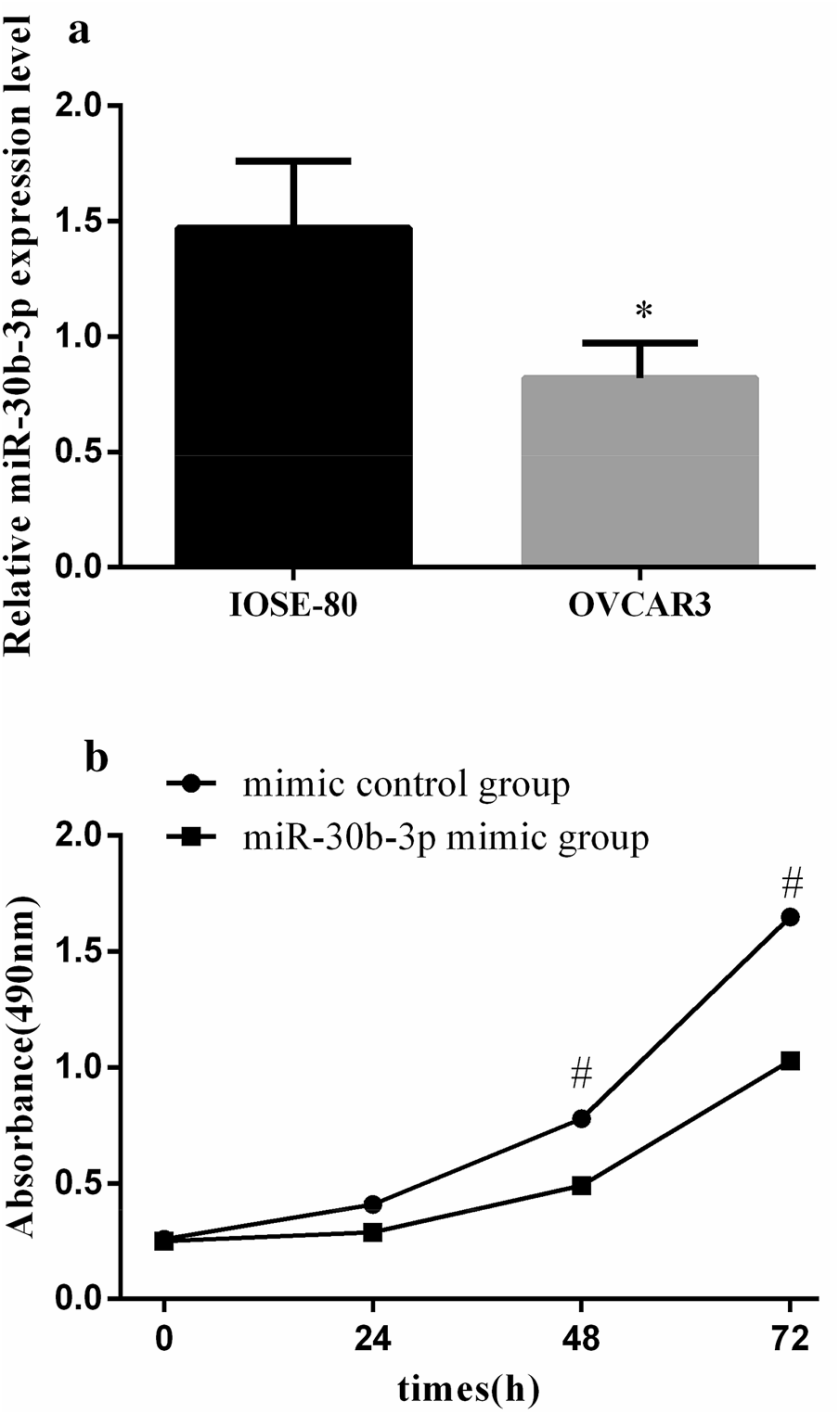
The relative expression level of miR-30b-3p in different ovarian cells and the effect of overexpression miR-30b-3p on OVCAR3 cells. **a** The result of qRT-PCR shown that the expression in OVCAR3 cells were significantly lower than those in IOSE-80 cells. *Mean P < 0.05 vs IOSE-80 cells. **b** Cell proliferation was suppressed after transfection of mir-30b-3p. ^#^Mean P < 0.05 vs mir-30b-3p mimic group

The expression of miR-30b-3p in human ovarian cancer epithelial cell line OVCAR3 cells was significantly lower than that in human normal ovarian epithelial cell line IOSE80 cells, and the difference was statistically significant (P < 0.05) (Fig. 1a).

### High expression of miR-30b-3p suppresses OVCAR3 proliferation

According to the cell transfection method, the cells were divided into mimic control group and mir-30b-3p mimic group. We investigated the effect of miR-30b-3p overexpression on cell proliferation by CCK-8 assay (Fig. 1b). The cell proliferation ability of mir-30b-3p mimic group decreased after 48 h, which was significantly lower than that of the mimic control group (P < 0.05), indicating that the high expression of mir-30b-3p could inhibit the proliferation of OVCAR3 cells.

### Transfection of miR-30b-3p can induced OVCAR3 cells apoptosis and slowed the growth speed of OVCAR3 cells

**Fig. 2:**
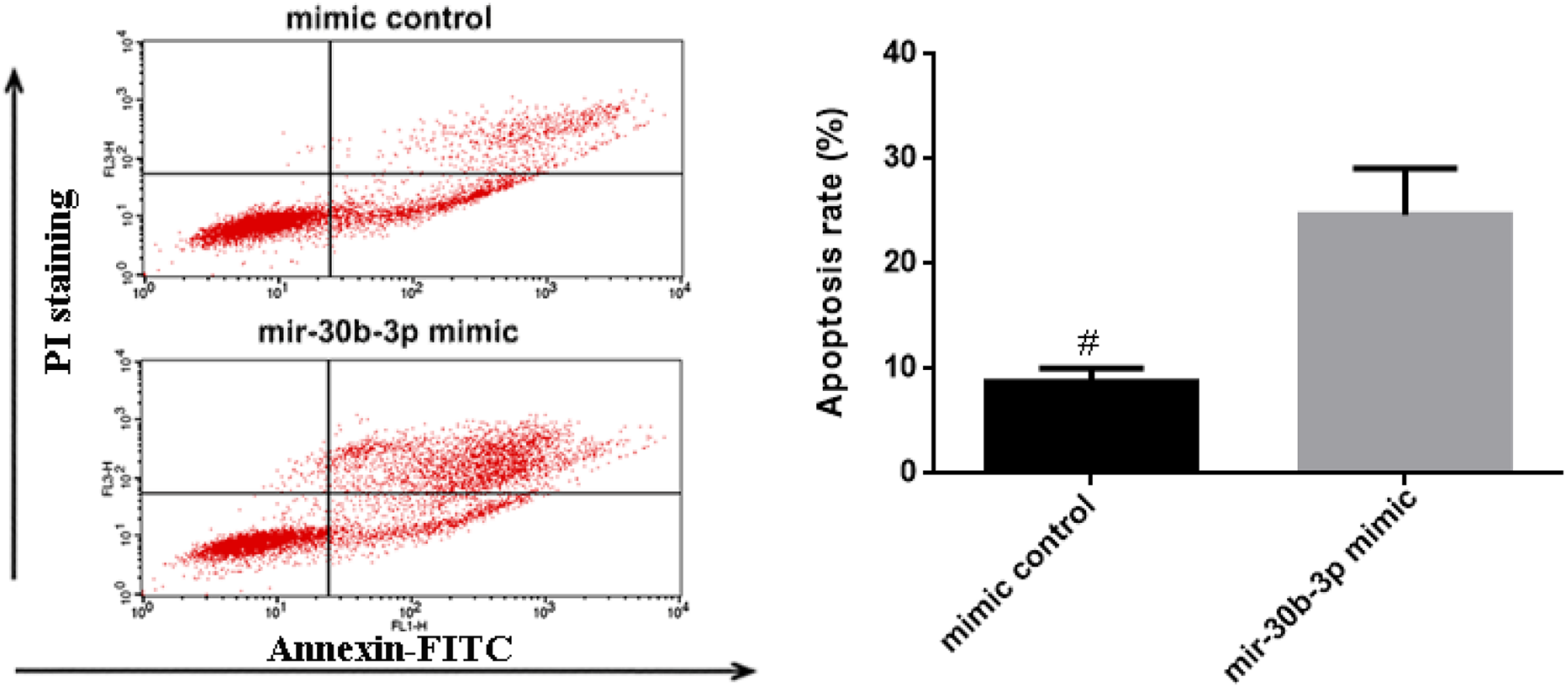
The effect of miR-30b-3p on OVCAR3 cells’ apoptosis. The apoptosis rate of mimic control group was 9.8%; the apoptosis rate of mir-30b-3p mimic group was 26.90%. ^#^Mean P < 0.05 vs mir-30b-3p mimic group

Flow cytometry was used to identify apoptosis and cell cycle in OVCAR3 cells (Fig. 2). The apoptosis rate of OVCAR3 cells transfected with miR-30b-3p were significantly higher than mimic control group cells (P < 0.05). And the growth rate of mir-30b-3p mimic group was significantly slower than mimic control group (P < 0.05). Cell cycle of OVCAR3 cells transfected with miR-30b-3p arrested in G0/G1 phase and S phase cells number decreased (Table 1). The result suggested that miR-30b-3p regulated the apoptosis and cell cycle of OVCAR3 cells.

**Table 1:** The division cycle of OVCAR3 cells (x ± s, %)

Group	G0/G1	G2/M	S
Mimic control group	44.13 ± 4.78^#^	14.52 ± 1.52	37.87 ± 3.42^#^
mir-30b-3p mimic group	69.16 ± 7.13	9.66 ± 1.45	15.94 ± 1.63

^#^Mean P < 0.05 vs mir-30b-3p mimic group

### Transfection of miR-30b-3p suppressed OVCAR3 cell migration and invasion

**Fig. 3:**
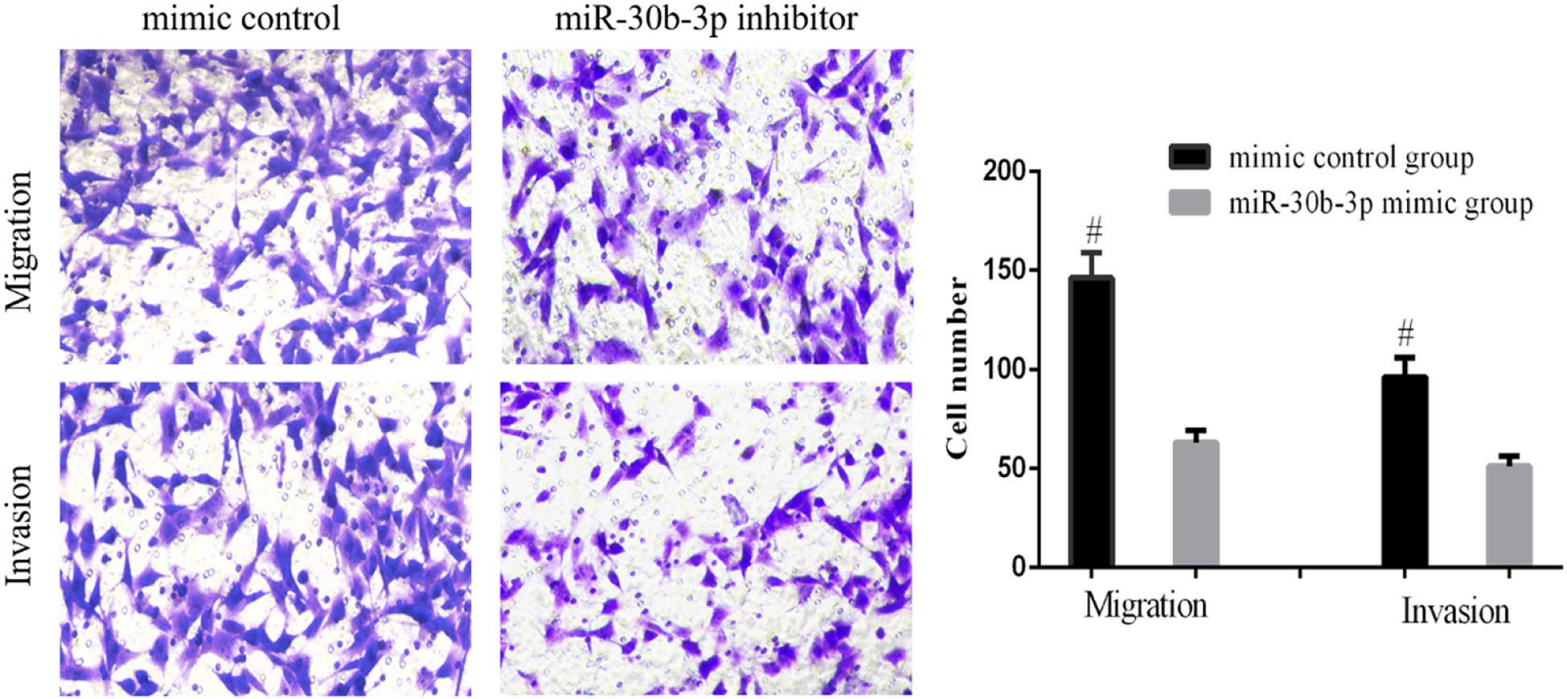
The effect of miR-30b-3p on OVCAR3 cell migration and invasion. High expression of miR-30b-3p notably inhibited the migration and invasion of OVCAR3 cell

Transfection with miR-30b-3p significantly reduced the number of OVCAR3 cells passing through the polycarbonate membrane and inhibited the invasion ability of OVCAR3 cells, with statistically significant differences compared with the mimic control group (P < 0.05) (Fig. 3).

### CTHRC1 is the target gene of miR-30b-3p

**Fig. 4:**
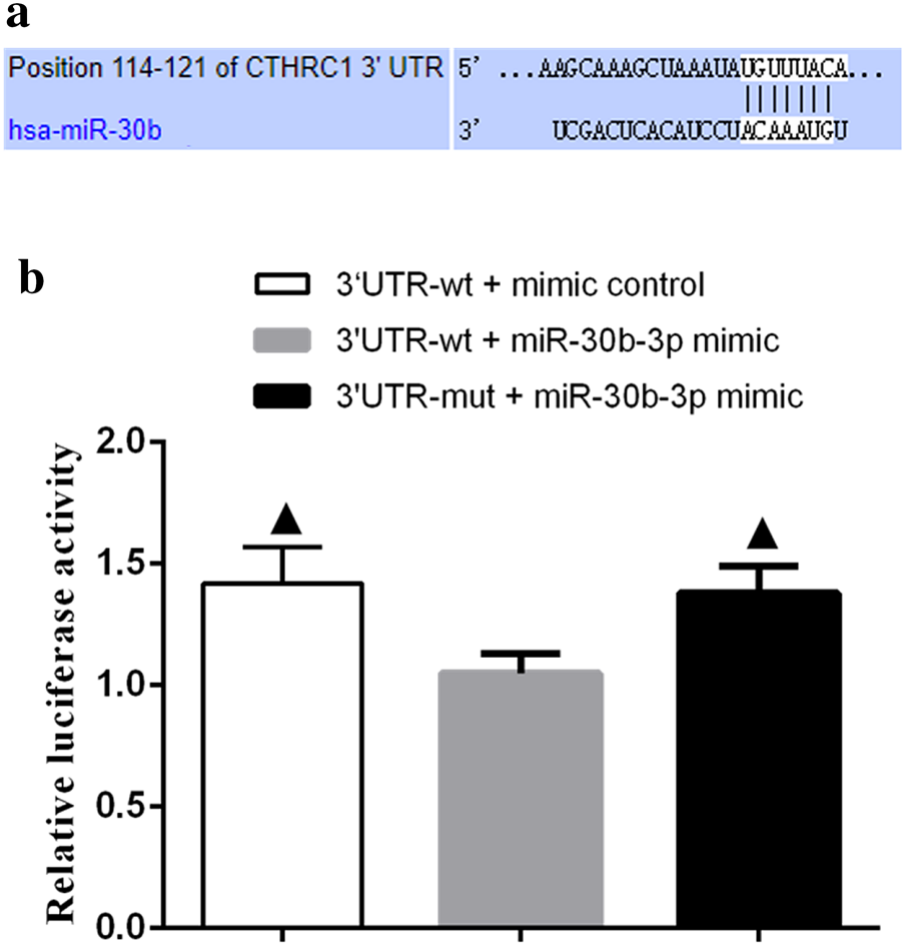
miR-30b-3p reduced the expression of CTHRC1 by targeting its 3′-UTR. **a** The potential binding site of miR-30b-3p was found in the 3′-UTR (114–121 bp) of CTHRC1 gene. **b** miR-30b-3p down-regulated luciferase activity of CTHRC1 wild-type 3′-UTR, but it had no effect on the luciferase activity of CTHRC1 mutant-type 3′-UTR. Black up-pointing triangle mean P < 0.05 vs 3′-UTR-wt + mir-30b-3p mimic

Bioinformatics analysis was performed using online tool TargetScan to identify potential targets of miR-30b-3p. The result shown that CTHRC1 mRNA contained a 3′-UTR element that was complementary to miR-30b (Fig. 4a). Hence, we cloned the CTHRC1 3′-UTR region containing this complementary site into the luciferase reporter vector. Compared with the mimic control group, the luciferase activity of OVCAR3 cells transfected with this structure and miR-30b-3p was significantly reduced. However, luciferase activity in cells transfected with reporter constructs containing suspected mir-30b-3p target mutations was not affected by mir-30b-3p co-transfection (Fig. 4b), indicating that miR-30b-3p targeted on the 3′-UTR of CTHRC1.

### MiR-30b-3p inhibits the expression of CTHRC1 protein

**Fig. 5:**
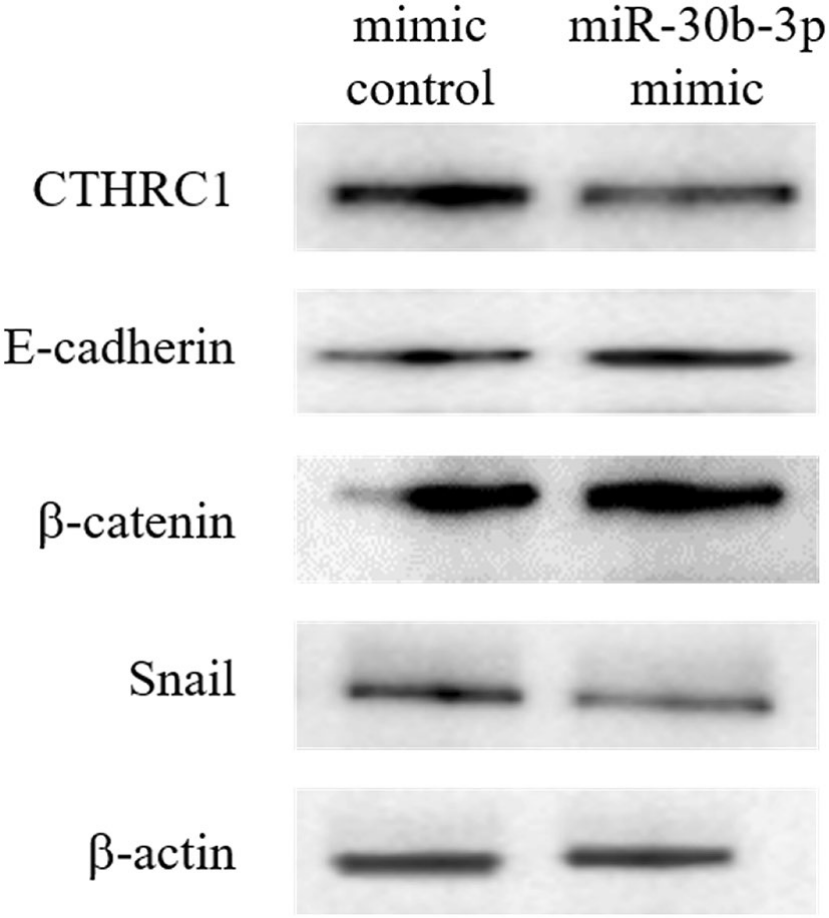
The protein expression levels of CTHRC1 and EMT-related genes were detected by Western-blot analysis in OVCAR3 cells. The expression of CTHRC1 in OVCAR3 cells transfected with mir-30b-3p was significantly lower than that in the control group. Furthermore, transfection of mir-30b-3p can result in increased expression of E-cadherin and β-catenin, while decreased expression of Snail

The protein expression of CTHRC1 in cell transfected with miR-30b-3p was significantly lower than mimic control group. Furthermore, transfection with miR-30b-3p resulted in upregulated expression of E-cadherin and β-catenin, and downregulated expression of Snail. The results indicated that miR-30b-3p increased the expression of E-cadherin and β-catenin by targeting CTHRC1 and ultimately inhibited the EMT process in ovarian cancer cells (Fig. 5).

## Discussion

There is growing evidence that miRNA are widely involved in cancer progression and suppression by regulating thousands of cancer-related genes [10]. MiRNAs have been confirmed that they have the potential for early cancer diagnosis and to predict therapeutic response and prognosis [11]. It is estimated that miRNAs control the activity of over 50% of all protein-coding genes in mammals [12] and are involved in the regulation of almost all cellular processes [13]. A lot of studies have shown that abnormal miRNA expression is associated with various human diseases including ovarian cancer [14, 15]. It has been identified that miR-30b upregulated in numerous malignancies including medulloblastoma [16], oral squamous cell cancer [17] and parathyroid carcinoma [18]. These results support the idea that mir-30b is an oncogene for these tumors. Nevertheless, miR-30b may also play a role as a tumor suppressor. Mir-30b expression has been found to be reduced in a variety of human cancers, such as gastric cancer [19], non-small cell lung cancer [20], and colorectal cancer [21, 22], indicating that miR-30b could suppress the invasion and migration of cancer cell and inhibit the proliferation of cancer cell. However, the mir-30 family has been less studied in ovarian cancer. Shi et al. [23] found that the high expression of miR-30 family was associated with the increase of OS/PFS in ovarian cancer, and the conclusion is that miR-30 family can be a reliable prognostic marker of ovarian cancer. The OncoLnc dataset showed that the increased expression level of miR-30b-3p was related to better OS [24]. These finding suggested that miR-30 family can act a tumor suppressor for ovarian cancer.

In this study, we first investigated the effect of mir-30b-3p on the biological function of ovarian cancer cells. The relative expression levels of miR-30b-3p in ovarian cancer cells line OVCAR3 and normal ovarian cells line IOSE-80 were detected by qRT-PCR, and the results showed that the expression of miR-30b-3p in OVCAR3 cells was lower than that in IOSE-80 cells. Mir-30b-3p was overexpressed in OVCAR3 cells by transfection. The overexpression of miR-30b-3p could inhibit the proliferation of OVCAR3 cells, slow down the cell division cycle, promote apoptosis, and reduce the migration and invasion of OVCAR3 cells. The results indicated that miR-30b-3p was a tumor suppressor gene for ovarian cancer.

Mir-30b-3p mainly affects the cell function of ovarian cancer by regulating its target genes. Bioinformatics analysis showed that mir-30b-3p was complementary to the 3′-UTR of CTHRC1. Therefore, the mutual regulation between the two was further verified by the luciferase reporting experiment, and it was found that miR-30b-3p could significantly inhibit the activity of luciferase, confirming that CTHRC1 was the target gene of miR-30b-3p.

CTHRCI is a secreted glycosylated protein, which was first found in the balloon injury model of rat and promotes cell migration by inhibiting collagen type I deposition [25]. Previous studies have shown that CTHRC1 is highly expressed in a variety of tumors and can be a prognostic factor for non-small cell lung cancer, liver cancer, gastrointestinal stromal tumor, pancreatic cancer, and colorectal cancer [26–29]. The mechanism of CTHRC1 in promoting tumor invasion and metastasis varies among different types of tumors. In colorectal cancer, CTHRC1 can enhance Erk phosphorylation, up-regulate MMP-9 expression, and promote extracellular matrix degradation, thereby promoting tumor cell invasion [30]. In addition, CTHRC1 promotes cell migration and adhesion through regulation of the Src/FAK signaling pathway in pancreatic cancer and integrinβ1 in hepatocellular carcinoma [31, 32]. Hou et al. [28] showed that increased expression of CTHRC1 in epithelial ovarian cancer can induce EMT in ovarian cancer cells, thereby promoting tumor cell invasion and metastasis. EMT is a process of molecular reprogramming and phenotypic change, which transforms polarized stationary epithelial cells into moving mesenchymal cells, thus resulting in malignant transformation and metastasis [33]. Studies have described that the dysregulated mir-30 family regulates multiple signaling pathways leading to tumor EMT, which plays a key role in tumor invasion and metastasis [9, 34]. On the other hand, some members of miR-30 family may also function as an EMT inhibitor, blocking the proliferation, invasion and metastasis of cancer cells [35]. In the study of Ye et al. [35], it was found that the expression level of mir-30 s in ovarian cancer cell lines was decreased after TGF-β induced EMT. On the contrary, overexpression of miR-30d could block EMT induced by TGF-β. It was confirmed that miR-30d directly bound to Snail’s 3′-UTR to inhibit its expression, thus inhibiting the EMT process.

To determine whether mir-30b-3p inhibits the migration and invasion ability of ovarian cancer cells through the regulation of EMT in OVCAR3 cells, this study tested the functional proteins related to EMT. The results showed that the overexpression of mir-30b-3p significantly reduced the expression of CTHRC1 protein, significantly increased the expression of E-cadherin and β-catenin, and significantly decreased the expression of Snail protein. This result indicated that mir-30b-3p inhibited the EMT process by binding to the 3′-UTR of CTHRC1, thereby inhibiting the migration and invasion ability of ovarian cancer cells. We first report that the direct regulation of CTHRC1 by miR-30b-3p.

## Conclusion

In summary, miR-30b-3p plays an anti-cancer role in ovarian cancer. The high expression of mir-30b-3p can inhibit the biological function of ovarian cancer cells. Mir-30b-3p can regulate the expression of EMT-related proteins and prevent the development of EMT in ovarian cancer cells by targeting and inhibiting the expression of CTHRC1. For the past few years, more and more evidences have shown that miRNAs have great potential in early detection, prognosis and chemotherapeutic sensitivity of ovarian cancer. Our study provides scientific basis for miR-30b-3p as a diagnostic or prognostic indicator for ovarian cancer. However, it is worth noting that the study has no conduct relevant clinical trials and animal model experiments. Hence, it is necessary to conduct further research upon our current research results.

## Data Availability

The data and materials of this experiment are available.

## Abbreviations

qRT-PCR: Quantitative reverse transcription polymerase chain reaction
CCK-8: Cell-counting kit-8
CTHRC1: Collagen triple helix repeat-containing 1
EMT: Epithelial–mesenchymal transformation
3′UTR: 3′-Untranslated region
PI: Propidium iodide
PBS: Phosphate buffer solution
